# Dual COX-2/5-LOX inhibitors from *Zanthoxylum simulans* inhibit gastric cancer cells by cross-mediating thyroid, estrogen, and oxytocin signaling pathways

**DOI:** 10.3389/fchem.2023.1287570

**Published:** 2024-01-10

**Authors:** Yong-Qiang Tian, Jing Liu, Peng Cheng, Jian Zou, Hui-Fang Xu, Xin-Hua Shi, Yi-Sheng Zhang, Ling Mei

**Affiliations:** ^1^ Department of Pharmacy, Wuhan Hospital of Traditional Chinese Medicine, Third Clinical Medical College of Hubei University of Chinese Medicine, Wuhan, Hubei, China; ^2^ Department of Acupuncture, Wuhan Hospital of Traditional Chinese Medicine, Third Clinical Medical College of Hubei University of Chinese Medicine, Wuhan, Hubei, China; ^3^ Department of Pharmacy, The Central Hospital of Wuhan, Tongji Medical College, Huazhong University of Science and Technology, Wuhan, Hubei, China

**Keywords:** *Zanthoxylum simulans*, ultrafiltration, COX-2 inhibitors, 5-LOX inhibitors, gastric cancer cells, hormone pathways

## Abstract

Cyclooxygenase 2 (COX-2) and 5-lipoxygenase (5-LOX) are overexpressed in gastric cancer cells, the dual inhibitors of which exhibit potential against metastasis and invasion with fewer side effects. To discover inhibitors targeting COX-2 and 5-LOX, we conducted ultrafiltration and enrichment calculation to screen candidates in quaternary alkaloids (QAs) from *Zanthoxylum simulans* through LC and LC-Q-TOF. For intensive peaks, peaks 19 (berberine) and 21 (chelerythrine) were observed as the most potent dual candidates and showed selective affinity to 5-LOX over COX-2. Peak 19 showed an enrichment at 4.36 for COX-2 and 22.81 for 5-LOX, while peak 21 showed an enrichment at 7.81 for COX-2 and 24.49 for 5-LOX. Molecular docking results revealed chelerythrine as a better dual inhibitor, showing time- and dose-dependent anti-proliferation against AGS cells. Bio-informatics strategies, such as Gene Expression Omnibus (GEO), Gene Ontology (GO), and Kyoto Encyclopedia of Genes and Genomes (KEGG), suggested that hormone pathways in gastric cancer cells might be mediated by chelerythrine. Further reviews and summaries helped outline the mechanisms by which COX-2/5-LOX inhibitors might promote apoptosis in gastric cancer cells via estrogen, thyroid, and oxytocin signaling pathways. Chelerythrine was also added to gastric cancer cells to verify the regulation of these three signaling pathways. As a result, significant calling back of thyroid-stimulating hormone receptor (TSHR), thyroid hormone α3 (TRα3), and thyroid hormone receptor β1 (TRβ1) and suppressing estrogen receptor α36 (ER-α36)–Src could benefit the anti-proliferation of chelerythrine. However, it was disappointing that regulation of estrogen receptor α66 (ER-α66), estrogen receptor β (ER-β), and oxytocin receptor (OTR) contributed inversely negative effects on anti-gastric cancer cells. At present, the integrative study not only revealed chelerythrine as the most potent dual COX-2/5-LOX inhibitor from QAs but also generally highlighted that comprehensive regulation of the estrogen, thyroid, and oxytocin pathway should be noted once gastric cancer cells were treated with inflammatory inhibitors.

## 1 Introduction

Gastric cancer is a malignancy associated with high rates of discovery and mortality in China, along with risk factors including *pylori* infection, smoking, hereditary factors, and heterogeneity (Wang et al., 2021). It is reported that proliferation, apoptosis, invasion, and migration of gastric cancer cells can be mediated by some pathways such as extracellular signal-regulated kinase/c-Jun (ERK/c-Jun), phosphate and tensin homolog/phosphoinositide 3-kinase/protein kinase B (PTEN/PI3K/AKT), and cAMP response element-binding protein 1/protein kinase B/glycogen synthase kinase-3 beta (CREB1/AKT/GSK-3β) (Lee et al., 2021; Zhao et al., 2021; Zhu et al., 2021). In addition, inflammation triggers these pathways to modulate the related cascades in carcinoma (Cheng et al., 2014; Su et al., 2023). For inflammatory targets, cyclooxygenase 2 (COX-2) and 5-lipoxygenase (5-LOX) are the key enzymes regulating the arachidonic acid pathway, which are overexpressed in gastric cancer cells and are beneficial in enhancing the process of cancer metastasis and invasion (Lin et al., 2021; Yang et al., 2023). Moreover, inhibitors of 5-LOX help with relieving the side effects of COX-2 on cardiovascular and asthmatic problems (El-Miligy et al., 2023). Thus, dual inhibitors targeting COX-2 and 5-LOX are regarded as potential and safer anti-proliferative candidates (Doiphode et al., 2023; Mukhopadhyay et al., 2023).

Hormones are necessary for the human body to maintain normal functional physiology; hence, their dysregulation can lead to a variety of diseases. In gender-related cancers, hormones play a vital role in carcinoma incidence, development, and mortality (Abdel-Rahman, 2020; Chlebowski et al., 2020). Carcinoma progression may be ameliorated by hormone replacement therapy in clinical trials (Abdel-Rahman, 2020; Chlebowski et al., 2020). In addition, epidemiological studies on gastric cancer not only revealed the discrepancy between men and women but also indicated the possible protective roles from estrogen signaling pathway in consideration of the higher incidence in men (Wang et al., 2014). As the most reported mediators in cancer, inflammation factors dysregulate the endocrine hormones, e.g., estrogen and oxytocin (Bowers and DeGraffenried, 2015; Kim et al., 2016). Meanwhile, it is still meaningful to figure out whether anti-inflammatory candidates may exhibit anti-cancer effects directly or indirectly via triggering hormone-related pathways.

In our previous study, total quaternary alkaloids (QAs) from *Zanthoxylum simulans* exhibited significant anti-proliferative effects on gastric cancer cells (Tian et al., 2017). The QA enrichment method was established and verified in a methodological investigation (Fan et al., 2019). Therefore, further studies should be carried out to further explore the bio-active substances and potential mechanisms. Because of multiple substances detected in QAs, it is not easy to figure out the bio-active ingredients and illustrate the potential mechanisms (Tian et al., 2022). Compound isolation combined with activity analysis is a traditional way to discover potential prodrugs, but the time-consuming method may not match the need of improving research efficiency (Tian et al., 2022). Ultrafiltration targeting of enzymes has been reported to fish potential bio-active candidates in recent years (Tian et al., 2022). For the exploration of mechanisms, some bio-informatics methods, including Gene Expression Omnibus (GEO), Gene Ontology (GO), and Kyoto Encyclopedia of Genes and Genomes (KEGG), are helpful to indicate possible mechanism assays based on public and massive data, which are reported to find the genes with significantly different expression, enrich possible pathways, and clarify the most valuable regulations (Shi et al., 2022; Bing et al., 2023; Quan et al., 2023). In combination with the predictive analysis, the following experiments are always used to confirm and convince the proposed genes, targets, and pathways (Shi et al., 2022; Bing et al., 2023; Quan et al., 2023).

Dual COX-2/5-LOX inhibitors are thought to be anti-gastric cancer candidates, but there is still a lack of studies that illustrate the mechanisms attributed to hormone signaling pathways. In this study, COX-2/5-LOX inhibitors from *Z. simulans* were screened through ultrafiltration to discover the potential anti-gastric cancer candidates. The most potent alkaloid, chelerythrine, was employed in *in silico* and bioinformatics analysis, to explore the potential inhibitory mechanisms for gastric cancer cells. We also detected the transcription and expression of major genes and targets in three hormone pathways to validate the indicated mechanisms.

## 2 Materials and methods

### 2.1 Materials and reagents

The original plant material was collected from Wuhan Botanical Garden, Chinese Academy of Sciences (Wuhan, China) in 2018 and was used to extract crude quaternary alkaloids. The National Institute for Food and Drug Control (Beijing, China) provided chelerythrine (CAS: 3895-92-9, 98.6%), magnoflorine (CAS: 2141-09-5, 99%), and berberine (CAS: 2086-83-1, 98%). Recombined enzymes, COX-2 and 5-LOX, were provided by Mingquan Guo from Wuhan Botanical Garden, Chinese Academy of Sciences. COX-2 and 5-LOX were originally purchased from Wuhan Antgene Biotechnology Co., Ltd. (Wuhan, China) and Cayman Chemical Co., Ltd. (Ann Arbor, MI, United States), respectively. WCX SPE columns were obtained from ANPEL Scientific Instrument Co., Ltd. (Shanghai, China). Cell lines of AGS (338141) and SGC-7901 were bought from BeNa Culture Collection (Beijing, China). The reagents and instruments used in this study, mainly including Src (Ab133283), the BCA protein assay kit (PICPI23223), glyceraldehyde-3-phosphate dehydrogenase (GAPDH) (Ab9485), RXR (GTX108728), Ham’s F-12K (Kaighn’s) Medium (F-12K), the SYBR Green PCR kit (K0223), oxytocin receptor (OTR) (#17198), Roswell Park Memorial Institute (RPMI) 1640 and TRIzol (1596-026), were obtained from Abcam (Cambridge, United Kingdom), Beijing Solarbio Science & Technology Co., Ltd. (Beijing, China), GeneTex (United States), CST (United States), Invitrogen (Carlsbad, CA, United States), Thermo Fisher Scientific (Waltham, MA, United States), and Gibco Life Technologies (Grand Island, NY, United States), respectively. Other reagents were of analytical grade and purchased from Sinopharm Chemical Reagent Co., Ltd. (Shanghai, China).

### 2.2 Ultrafiltration

The deposited plant material of *Z. simulans* in our previous study (No. 2018ZS001) was extracted to generate crude alkaloids in this study (Tian et al., 2017). According to the established method, total QAs were enriched from the crude alkaloids on WCX cartridges (Fan et al., 2019). Ultrafiltration was conducted based on the revised approach in our previous study (Tian et al., 2022). After being dispersed with PBS (pH 7.4), the recombined enzymes, COX-2 and 5-LOX, were separated into 5 U per aliquot and stored in EP tubes. For each enzyme, 10 μL of an aliquot was placed in boiling water for 30 min to prepare the inactive enzyme and used as the negative control. Here, 10 μL of aliquots was selected to mix with QAs. After incubating at 37°C for 30 min, the mixture was centrifuged to filtrate unbound substances (YM-30 membrane for COX-2, 30 kD; YM-10 membrane for 5-LOX, 10 kD). By rinsing with PBS three times, the conjugation of the enzyme–alkaloid was denatured with methanol and further centrifuged to separate the potential bioactive alkaloids. The final eluent in ultrafiltration was collected and dried under nitrogen. The residue was dissolved with 5% methanol to produce samples for LC and LC-Q-TOF-MS.

### 2.3 LC and LC-Q-TOF analysis

Samples generated by ultrafiltration were analyzed under almost the same chromatographic conditions as in our previous study (Tian et al., 2022). A 2.5 μm Waters XSelect CSH column (2.1 mm × 150 mm) was used in gradient elution at 30°C. The gradient elution was conducted with 0.1% formic acid (A) and acetonitrile (B) as follows: 0–5 min, 5% (B); 5–40 min, 5%–40% (B); 40–60 min, 40%–90% (B). Chromatograms at 280 nm were recorded when 2 μL of the sample was injected into a Waters Arc 2998. The peaks and peak area were recorded to indicate enrichments for enzymes. To figure out the detected alkaloids in QAs, MS/MS spectrums were obtained from an Agilent 1290 combined 6530 mass spectrometer (Tian et al., 2022). Using full scan mode from m/z 100 to 1,000, the MS/MS spectrums of collision energy at 10, 20, and 40 V were obtained under the default setting of mass conditions, mainly including capillary voltage at 3,500 V, gas temperature at 300°C, fragmentor voltage at 175 V, and skimmer voltage at 65 V. Published literature and standards were used to identify the alkaloids by comparing the retention time and MS/MS data.

### 2.4 Cell culture and anti-proliferation analysis

After being cultured in RPMI-1640 medium at 37°C with 10% FBS, AGS cells in the logarithmic phase were prepared by trypsin and adjusted to 1 × 10^4^/mL. The cells were added to a 96-well plate at 100 μL/well in triplicate and treated with chelerythrine at 0, 1, 2, 5, 10, 20, 40, 80, and 160 μg/mL for 0, 24, 48, and 72 h. CCK-8 was applied to determine the anti-proliferative activity. DMSO was used as the blank. The data of OD values were observed under a microplate reader DNM-9620 (Beijing Pulang New Technology Co., Ltd., Beijing, China). The inhibitory rate (%) = (1-OD_Che_/OD_blank_) × 100%, where OD_Che_, and OD_blank_ are the values of chelerythrine and the blank.

### 2.5 Molecular docking

Molecular Operating Environment (MOE, v2019.01, Chemical Computing Group, Montreal, Canada) was used to simulate the interaction between selected alkaloids and targets (Tian et al., 2022). The 2D structures of peak 5 (magnoflorine), 19 (berberine), 21 (chelerythrine), and the positive control (rofecoxib and nordihydroguaiaretic acid [NDGA]) were imported to establish a compound database. Then, 3D conformations were prepared to obtain a stereo-structure database. Crystallized structures of the enzymes were downloaded from RCSB PDB (COX-2 PDB: 5KIR; 5-LOX PDB: 6N2W) based on previous studies (Orlando and Malkowski, 2016; Gilbert et al., 2020). After removing water and adding hydrogen, one chain containing a ligand was kept and optimized for minimum energy conformation. Molecular docking was conducted in the active pocket. London dG scores were produced by using the Triangle Matching method to dock at 30 preferential poses. In addition to energy scores, the interactions of residues, energy, and bonds were displayed, compared, and discussed to evaluate the ligand–target interactions.

### 2.6 Differentially expressed genes screening

The source data were downloaded from the GEO database (GSE118916; https://www.ncbi.nlm.nih.gov/). Differentially expressed genes (DEGs) between gastric cancer and normal negative control samples were found using the R package “limma.” *p* < 0.05 and log2 fold change (FC) > 1 were set as the threshold to select differently expressed genes.

### 2.7 GO enrichment assay

Metascape (https://metascape.org) was used to conduct GO enrichment of DEGs. The clustered genes related to hormones were picked up and input into STRING (https://cn.string-db.org/) to deduce the network among these genes. The network was further filtrated and visualized in Cytoscape (version 3.7.2).

### 2.7 KEGG enrichment assay

The Traditional Chinese Medicine Systems Pharmacology Database and Analysis Platform (https://old.tcmsp-e.com/tcmsp.php) was used to obtain potential targets of chelerythrine. The genes of the targets were collected from UniProt (https://www.uniprot.org/). After searching GeneCards (https://www.genecards.org/), gastric cancer-related genes were obtained. Common genes in target-related and gastric cancer-mediated were selected to conduct KEGG enrichment via “R”.

### 2.8 Quantitative real-time PCR assay

Gastric cancer cells, AGS and SGC-7901, were treated with chelerythrine and used in RNA extraction with TRIzol reagents (Invitrogen, Carlsbad, CA, United States). cDNA was synthesized by following the instructions of a Revert Acid First Strand cDNA Synthesis Kit (Fermentas, Thermo Fisher Scientific, United States). Then, primers and cDNA were mixed. Amplification was continued in the RT-qPCR using the SYBR Green PCR kit (Thermo Fisher Scientific, United States) on an Applied Biosystems (ABI) machine (Applied Biosystems, United States). Ct values were recorded, calculated, and displayed as 2-ΔΔCq. GAPDH was set as the control and used to normalize the relative mRNA transcription. The primers were F: 5′- TCC​AAG​TTC​CAG​GAT​ACT​C -3′ and R: 5′- GCC​CAT​TAT​GTC​TTC​ACA​C -3′ for thyroid-stimulating hormone receptor (TSHR), F:5′- GCAGAAAGTGAGCAAGAG -3′ and R: 5′- TGATGTCCGTGAAGTACC -3′ for thyroid hormone α3 (TRα3), F:5′- CGG​GTA​CTT​GAA​ACT​ATT​G -3′ and R: 5′- AGG​TGG​TCG​TAT​TAT​CTT​G -3′ for thyroid hormone receptor β1 (TRβ1), F:5′- GCAAGATCGCTAGAACAC -3′ and R: 5′- GAGCATCCCTCTTTGAAC -3′ for estrogen receptor α36 (ER-α36), F:5′- TCTACAGGCCAAAT TCAGATAATCGA -3′ and R: 5′- CCC​TCA​CAG​GAC​CAG​ACT​CCA​TA -3′ for estrogen receptor α66 (ER-α66), F:5′- GCAAGATCGCTAGAACAC -3′ and R: 5′- GAGCATCCCTCTTTGAAC -3′ for estrogen receptor β (ERβ), F:5′- GGTGATGGCGTATGTTTG -3′ and R: 5′- TTGTCTGATGGCTGAGTC -3′ for the OTR, and F:5′- CAC​CCA​CTC​CTC​CAC​CTT​TG -3′ and R: 5′- CCA​CCA​CCC​TGT​TGC​TGT​AG -3′ for GAPDH.

### 2.9 Western blot assay

Gastric cells, AGS and SGC-7901, were lysed with RIPA buffer (Solarbio, Beijing, China) to extract the total protein. The BCA protein assay kit (Thermo Fisher Scientific, United States) was used to quantify the protein content. A measure of 20 μg crude protein was loaded and separated in SDS-PAGE gel. After transferring PVDF membranes and blocking with milk, primary antibodies were added to probe the membranes overnight at 4°C. An HRP-conjugated second antibody was added to enhance the protein bond by using an enhanced chemiluminescence kit (ECL) (Millipore, United States). As an endogenous protein, GAPDH was selected to normalize the proteins of retinoid X receptor (RXR), oxytocin receptor (OTR) and Src.

### 2.10 Statistical analysis

For statistical analysis, the data were presented as mean ± standard deviation (SD). ANOVA and variance were used to evaluate the quantitative data of genes and target protein expression by using SPSS (Version 16.0). A *p*-value < 0.05 (marked as *) or 0.01(marked as **) was considered statistically significant.

## 3 Results and discussion

### 3.1 Screening potential dual COX-2/5-LOX inhibitors

**FIGURE 1:**
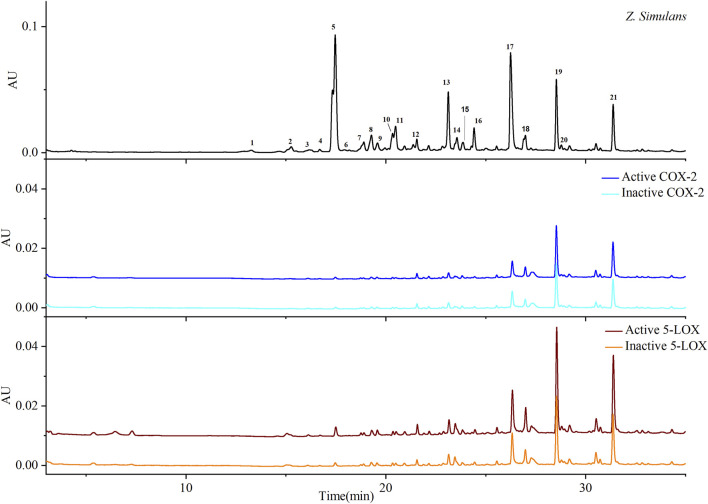
Chromatograms based on ultrafiltration. COX-2 and 5-LOX enzymes were used to screen potential bio-active substances. Colors, blue and cyan, were used to indicate active and inactive samples produced by COX-2 enzyme, respectively, while wine and orange indicate active and inactive 5-LOX, respectively.

QAs were enriched from the crude material of *Z. simulans* (*Z. simulans*) based on established methods (Fan et al., 2019). Under separation conditions (Tian et al., 2022), 21 peaks were separated and detected in this study, as shown in Figure 1 and Table 1, and peaks 5, 13, 17, 19, and 21 were the most obvious and intensive peaks. Although some discrepancies were noted by comparing with the chromatograms obtained in a previous study, similarities remained the most important essential part, given the batch source of SPE cartridges, reagents, and some ambient conditions. In the following ultrafiltration for COX-2 and 5-LOX, enrichment factors (EFs) were used to indicate the affinity between enzymes and potential ligands. $\text{EFs}=\frac{A_{a}-A_{i}}{A_{QAs}}\times 100\%$ , where $A_{a}$ , $A_{i}$ , and $A_{QAs}$ indicate the peak areas of samples incubated with active and inactive enzymes and QAs, respectively. As the results in Table 1, the alkaloids of peaks 18, 19, 20, and 21 not only showed the most affinity to COX-2 with EFs at 3.44, 4.36, 5.07, and 7.81, respectively, but also to 5-LOX with EFs at 16.45, 22.81, 19.69, and 24.49, respectively. Unlike peaks 19 and 21, other intensive peaks, i.e., peaks 5, 13, and 17, showed EFs at 0.05, 0.32, and 0.08 for COX-2 and 1.58, 2.00, and 3.85 for 5-LOX, respectively. In consideration of peak area and EFs, peaks 19 and 21 were more likely to be the bioactive dual inhibitors.

**TABLE 1:** EFs and SI of potential inhibitors of COX-2 and 5-LOX from *Zanthoxylum simulans*.

Peak No.	Rt (min)	M^+^ (m/z)	EFs (%)		SI	Identification
			COX-2	5-LOX		
1	13.273	274.1463	0.00	0.00	—	2H-Pyrano[2,3-b]quinoline or its isomers
2	15.264	286.1464	0.00	1.39	—	7-Methoxyl higenamine
3	16.180	344.1888	0.00	4.99	—	Isotembetarine or xylopinidine
4	16.691	328.1569	0.00	0.28	—	Unidentified
5	17.466	342.1729	0.05	1.58	—	Magnoflorine
6	17.923	312.1262	0.00	0.00	—	Unidentified
7	18.900	330.1730	1.40	0.89	—	Reticuline
8	19.279	358.2042	0.21	3.65	—	8-Methoxy-isotembetatrine
9	19.582	314.1775	0.38	6.40	—	Magnocurarine or its isomers
10	20.339	356.1885	0.02	1.66	—	N-Methyltetrahydrocolumbamine
11	20.487	356.1855	0.10	1.56	—	Menisperine
12	21.551	304.1569	1.26	13.82	—	Unidentified
13	23.125	356.1885	0.32	2.00	—	Xanthoplanine
14	23.558	368.1523	0.35	8.30	—	10-Hydroxy-2,3,9,12-tetramethoxy-jatrorrhizine
15	23.862	354.1731	1.26	5.24	—	N-Methylcanadine or its isomers
16	24.416	342.2098	0.09	1.89	—	Unidentified
17	26.243	354.1729	0.08	3.85	—	N-Methylcanadine or its isomers
18	26.984	354.1730	3.44	16.45	4.79	N-Methylcanadine or its isomers
19	28.535	336.1256	4.36	22.81	5.23	Berberine^a^
20	28.782	334.1101	5.07	19.69	3.88	Isoterihanine
21	31.377	348.1255	7.81	24.49	3.14	Chelerythrine^a^

EFs, enrichment factors; SI, selectivity index.

^a^ Identified by comparing the Rt and MS/MS spectrum with standards.

—: not detected.

As shown in Table 1, most ligands exhibited better EFs to 5-LOX than to COX-2. The EFs of peaks 18, 19, 20, and 21 were obvious and included in calculating the selective index (SI). SI= $\frac{{EFs}_{5-LOX}}{{EFs}_{COX-2}}$ , where ${EFs}_{5-LOX}$ and ${EFs}_{COX-2}$ represent the enrichment factors of these potential ligands to 5-LOX and COX-2, respectively. As a result, the SI of peak 19 was proposed at 5.23, while for peak 18 at 4.79, for peak 20 at 3.88, and for peak 21 at 3.14. These four peaks had similar SIs, indicating almost the same role in mitigating the side effects due to COX-2 inhibition.

In the arachidonic acid pathway, COX-2 and 5-LOX are commonly emphasized because of their roles in regulating prostaglandins (PGs) and leukotrienes (LTs), which mediate inflammation directly via participating in multiple pathways (Mukhopadhyay et al., 2023). Only inhibition of COX-2 might lead to the accumulation of LTs and further respiratory side effects (El-Miligy et al., 2023). Selective and dual inhibitors are considered safer candidates. Ultrafiltration combined LC-Q-TOF-MS/MS supported that peak 19 (berberine) and peak 21 (chelerythrine) from QAs might be the potential dual inhibitors in this study. In comprehensive consideration of enrichment, selectivity, and relative content, we were inclined to consider chelerythrine as the most bio-active and safer dual COX-2/5-LOX inhibitor among QAs.

### 3.2 Alkaloid identification

QAs were analyzed by LC-Q-TOF to provide MS/MS data for compound identification. As shown in Table 1, most alkaloids were identified by comparing their retention time, molecular weight, and MS/MS spectrum with those obtained in a previous study (Tian et al., 2022). As the most potential dual inhibitors in this study, peak 19 and peak 21 were authentically identified as berberine and chelerythrine, respectively, by comparing MS/MS spectrums with standards.

### 3.3 Molecular docking

To better understand the affinity results in ultrafiltration, molecular docking was conducted by MOE to speculate on the interactions between potential inhibitors and targets. Rofecoxib (Vioxx) and NDGA were reported to robustly inhibit the catalytic ability of COX-2 and 5-LOX and were selected as the positive control (Orlando and Malkowski, 2016; Gilbert et al., 2020). As the most intensive peak but with weak EFs among QAs, magnoflorine was selected as the negative control.

**FIGURE 2:**
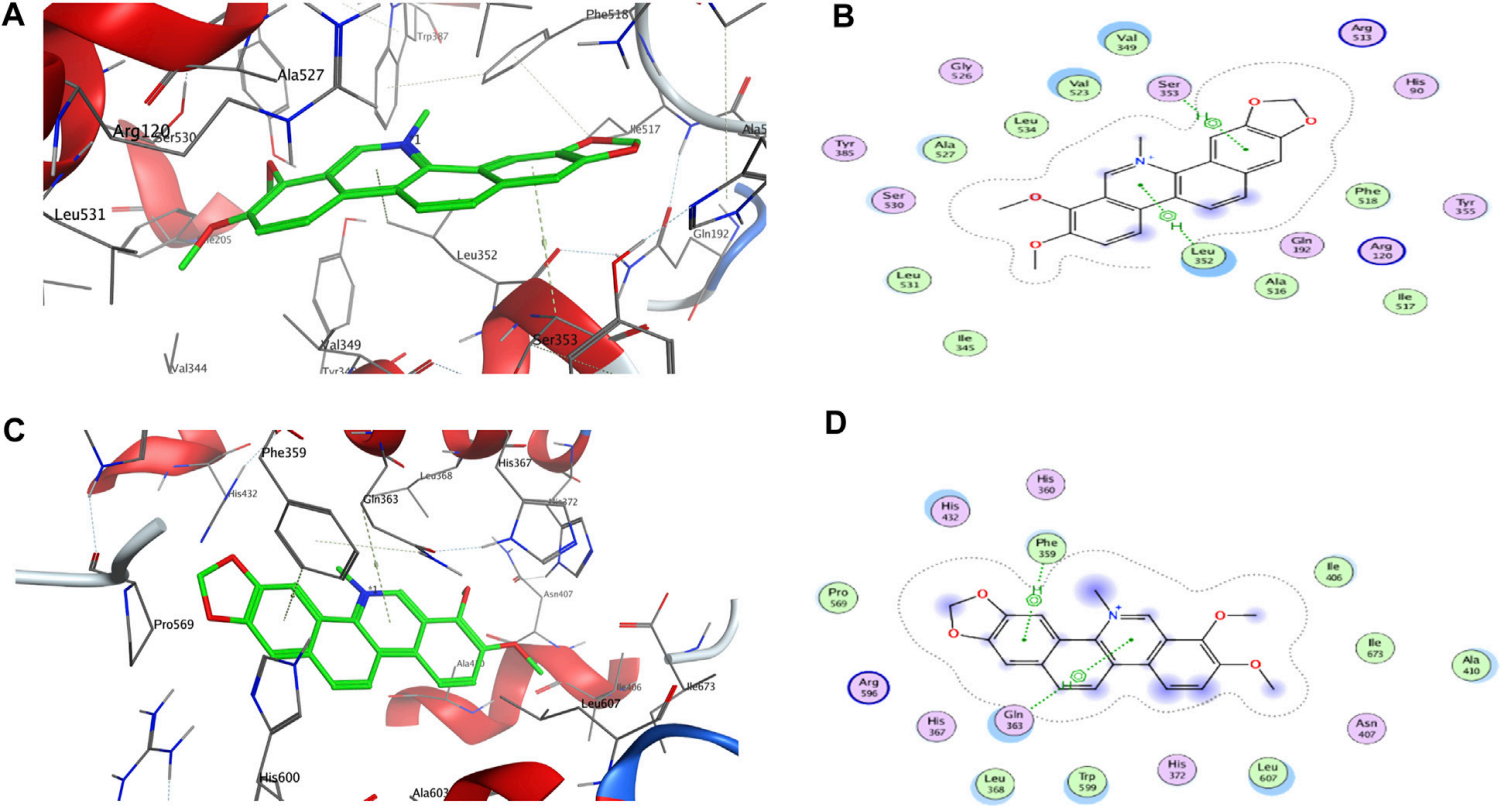
3D and 2D interactions between targets and chelerythrine. Molecular docking was used to simulate the 3D **(A)** and 2D **(B)** interactions between COX-2 and chelerythrine, while 3D **(C)** and 2D **(D)** for 5-LOX and chelerythrine, respectively.

As shown in Table 2, rofecoxib exhibited the best energy score than the alkaloids from QAs. Residues of COX-2, Leu 352 (−0.5), Arg 120 (−12.0), Arg 513 (−5.5), and Ala 527 (−0.7) interact with rofecoxib. Chelerythrine and berberine acted on Leu 352 and Ser 353 through an arene–H bond, as shown in Figure 2, while magnoflorine did not form a direct force bond. Without many polar moieties compared with rofecoxib, chelerythrine, and berberine showed weaker interaction energies and energy scores. Leu 352 in COX-2 was the common interaction residue for the two alkaloids, the forces of which were mainly attributed to the aromatic rings in the skeleton.

**TABLE 2:** Results of docking with COX-2 and 5-LOX.

Compounds	COX-2		5-LOX	
	Energy score	Interactions with residues (ES, kal/mol)	Energy score	Interactions with residues (ES, kal/mol)
Chelerythrine	−6.81	Leu 352 (−0.5) and Ser 353 (−0.7)	−5.71	Phe 359 (−0.7) and Gln 363 (−1.0)
Berberine	−6.87	Leu 352 (−0.5) and Ser 353 (−0.5)	−5.65	His 372 (−0.6) and Phe 359 (−0.7)
Magnoflorine	−6.10	--	−5.40	His 372 (−0.6)
Rofecoxib^a^	−7.58	Leu 352 (−0.5), Arg 120(-12.0), Arg 513 (−5.5), and Ala 527(-0.7)	—	—
Nordihydroguaiaretic acid^b^	—	—	−6.22	Asn 407 (−2.5), His 600 (−1.3), Arg 596 (−1.0), and Phe 359 (−0.7)

ES, Energy Score in docking.

^a^ Reported as a selective inhibitor to COX-2 in the literature.

^b^ Reported as an inhibitor to 5-LOX by lodging in the active cavity in the literature.

–: Not detected. ‐‐: No interactions observed in this docking.

For 5-LOX, as shown in Table 2, nordihydroguaiaretic acid showed an arene–hydrogen bond (H bond) and hydrogen bond with Asn 407 (−2.5), His 600 (−1.3), Arg 596 (−1.0), and Phe 359 (−0.7), resulting in the highest energy score among the candidates. As shown in Figure 2, chelerythrine formed an arene–H bond with Phe 359 (−0.7) and Gln 363 (−1.0), while berberine formed an arene–H bond with His 372 (−0.6) and Phe 359 (−0.7), and magnoflorine formed an arene–H bond with His 372 (−0.6). Chelerythrine had a better energy score and stronger interaction energy than berberine, and an aromatic ring contributed to the interactions with Gln 363 and Phe 359. With the backbone atoms not in a flat plane, magnoflorine exhibited the least interactions with the 5-LOX enzyme. Taken together, the docking results supported chelerythrine as the most potent dual inhibitor among the QAs.

Molecular docking is a well-known *in silico* method to simulate interactions between targets and potential inhibitors. Once lodged in the cavities of enzymes, inhibitors might interact with residues in the stereo-cavities and lead the stereo-structures to change through forming forces in experiments, including ion bonds, H-bonds, van der Waals force, and hydrophobic interactions (Tian et al., 2022). Even though the alkaloids in this study contained quaternary N atoms in the skeleton, an ion bond and hydrogen bond were still not obviously observed. Except for hydrophobic interactions owing to aromatic groups, van der Waals forces were also observed. As shown in Figure 2B, some atoms in the aromatic ring were exposed to the residues of Leu 352, Val 349, Val 523, and Ala 527, which were further recognized as backbone donors to backbone acceptors, Arg 120 and Arg 513, for COX-2. For docking interactions shown in Figure 2D, residues His 432, Gln 363, Leu 368, Trp 599, Leu 607, Ala 410, Ile 406, and Phe 359 were exposed to chelerythrine, while the backbone donation of Leu 607, Trp 599, Leu 368, Phe 359, Pro 569, Ala 410, Il673, and Il406, but not His 432 and Gln 363, were accepted by Arg 596. The exposure of chelerythrine and enzymes offered the possibility to transform the backbones and sidechains in the stereo-cavity when inhibitors were placed in the active pockets. In addition to forming direct bonds, the energy from stereo-structure conformations also contributed to the EFs in ultrafiltration. Polar moieties always form hydrogen bonds in docking cavities. On the contrary, greasy groups and interactions of alkaloids in this study might play a crucial role in affinity. Thus, it was probably the best strategy to modify chelerythrine structure by adding polar moieties to improve the interactions with targets but keeping the skeleton of the alkaloid still.

### 3.4 Chelerythrine inhibited AGS cells dose- and time-dependently

**FIGURE 3:**
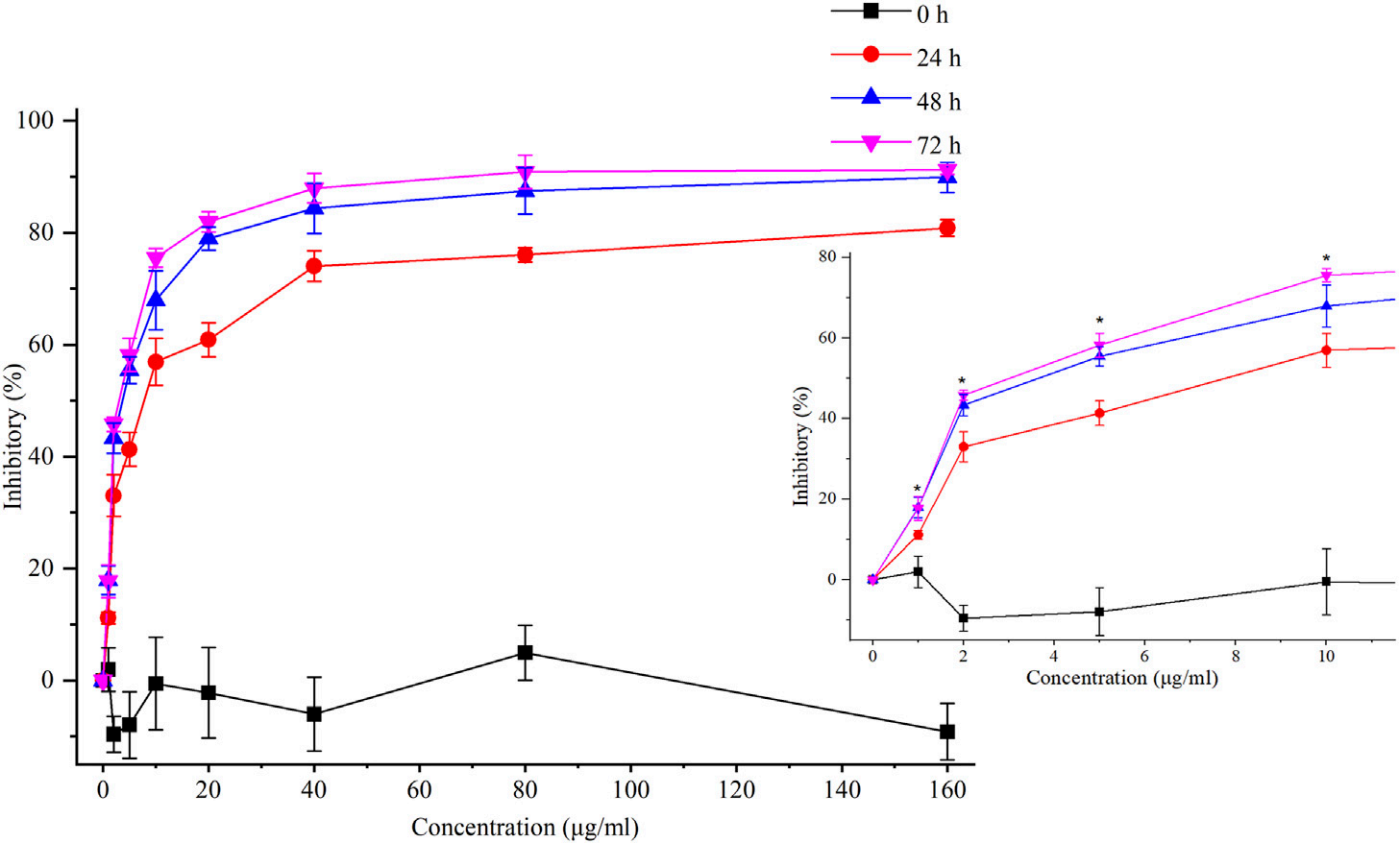
Inhibitory effects of chelerythrine on AGS cells. AGS cells were incubated with chelerythrine (0, 1, 2, 5, 10, 20, 40, 80, and 160 μg/mL) for 0, 24, 48, and 72 h. Then, inhibitory rates were calculated based on the data obtained from CCK-8 assay (*n* = 3). One-way ANOVA was used to indicate significance. *p < 0.05 vs. data at 24 h.

In our previous study, chelerythrine exhibited anti-gastric cancer potential via inhibiting migration, adhesion, invasion, and promoting apoptosis (Tian et al., 2022). To further explore the anti-proliferative effects on gastric cancer cells (AGS), chelerythrine was prepared in gradient concentrations at 0, 1, 2, 5, 10, 20, 40, 80, and 160 μg/mL. Chelerythrine was added to AGS cells for 0, 24, 48, and 72 h in the CCK-8 assay to disclose the anti-cancer efficacy. As shown in Figure 3, inhibitory rates increased with the treatment time and concentration. For the same treatment time, the inhibitory curve rose sharply at 0–10 μg/mL, which made it the most sensitive concentration range. For the same treatment concentration, a time-dependent suppression was recognized. The inhibitory curves at 48 and 72 h were closer than those at 24 h. One-way ANOVA analysis revealed significant differences between 48 h and 24 h at 1, 2, 5, and 10 μg/mL (*p* < 0.05), which were not observed between 72 h and 48 h (*p* > 0.05). Thus, the half-maximal inhibitory concentration (IC_50_) was determined at 4.09 μg/mL (11.75 μM, [M]+:348.1255) based on the data at 48 h. In addition to the IC_50_ value of chelerythrine at 11.81 μM obtained in the sulforhodamine B (SRB) assay in our previous study (Tian et al., 2022), we reported that chelerythrine showed anti-proliferative effects on AGS cells in a dose- and time-dependent manner. To maintain utmost consistency in results from different studies, chelerythrine was diluted in a series of concentrations at 0, 2.95, 5.91, and 11.81 μM, corresponding to 0, 1.03, 2.06, and 4.11 μg/mL, respectively, which were used in the following experiments.

### 3.5 Inflammation mediated hormone signaling pathways in gastric cancer

**FIGURE 4:**
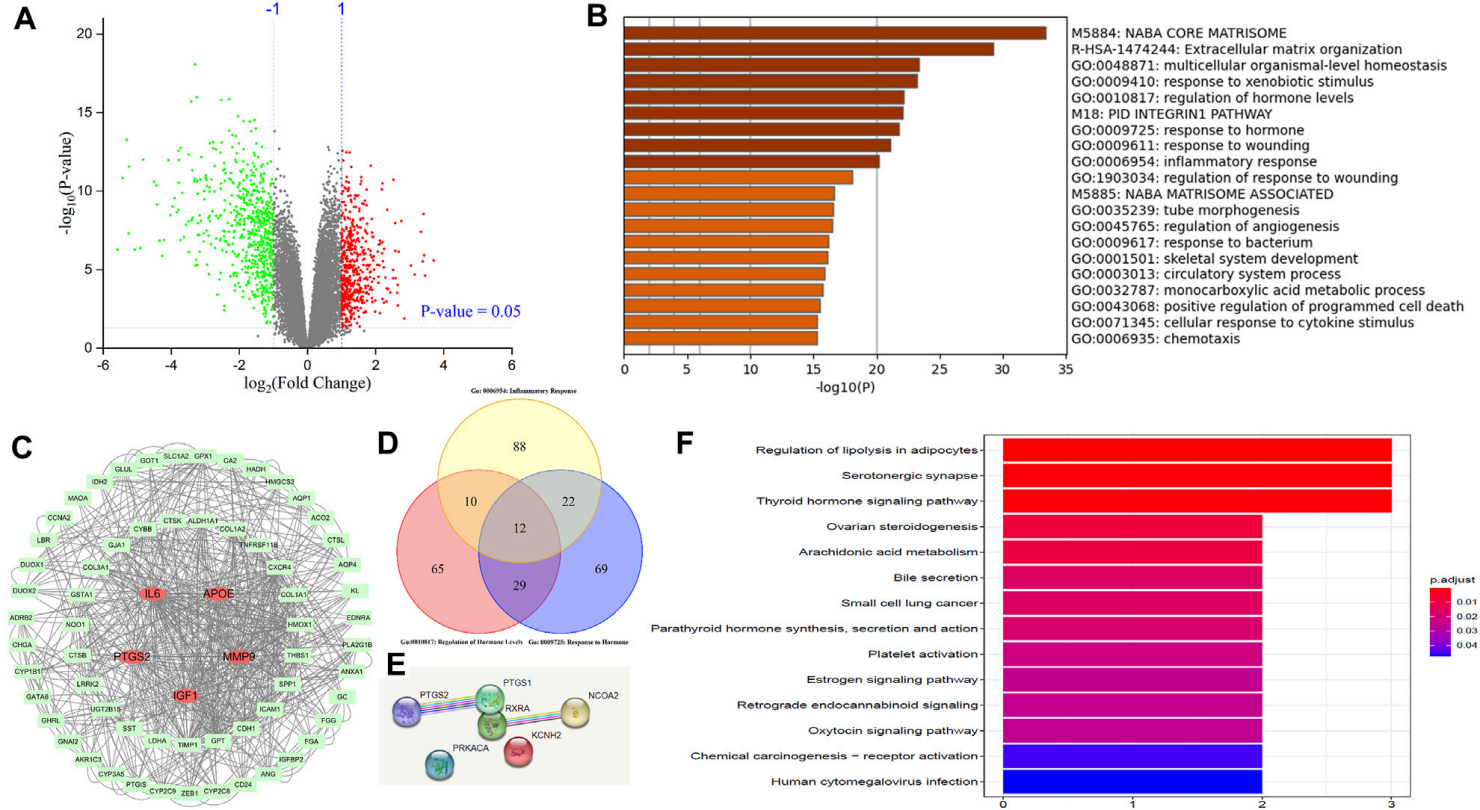
Potential pathways between gastric cancer cells and chelerythrine were indicated based on GEO, GO, and KEGG enrichment. **(A)** Volcano plot in GEO analysis. The upregulated genes were represented as red dots, while downregulated genes are indicated in green. **(B)** GO enrichment of the upregulated and downregulated genes. Enrichment results were generated through inputting significantly expressed genes into Metascape. **(C)** Network of major hormone-related genes in GO enrichment. Original data in GO enrichment were screened to obtain hormone-related genes and used to further reconstruct the network among these genes in STRING. Then, major genes were visualized in Cytoscape. The five most important genes are in ellipse shape (red), while others are shown in round rectangle shape (light green). **(D)** Venn diagram of genes related to hormones and inflammation in GO enrichment. **(E)** Network of potential chelerythrine targets. **(F)** Potential pathways mediated by chelerythrine were indicated by KEGG enrichment.

To mine the most important mechanisms of gastric cancer, the GEO was used to find DEGs (Supplementary Table S1) based on GSE118916 from the GEO database. As shown by the volcano plot in Figure 4A, the transcription of 1,143 genes was found to be significantly regulated under the threshold of |log2 FC| > 1 and FDR < 0.05. Of these, 511 genes were upregulated and 497 were downregulated. The DEGs were collected and input into Metascape to conduct GO enrichment. The most enriched 20 clusters are displayed in Figure 4B (Supplementary Table S2). Then, we shed light on Go: 0010817 and Go: 0009725, which stressed the importance of hormone response and regulation in gastric cancer. The genes of these two biological processes were collected and used to indicate their correlations in STRING, which were further exported and employed to screen hub genes considering betweenness centrality, closeness centrality, and degree. As shown in Figure 4C, the filtered major genes were re-visualized in Cytoscape 3.7.2. Finally, we screened out 67 genes with the most nodes, five of which, namely, interleukin-6 (IL-6), insulin-like growth factor-1 (IGF-1), matrix metalloproteinase-9 (MMP-9), prostaglandin G/H synthase 2 (PTGS2, also known as COX-2), and apo lipoprotein E (APOE), were more potent and are highlighted in red. The Venn diagram shown in Figure 4D was produced based on the genes of Go: 0010817, Go: 0009725, and Go: 0006954. Common factors, IL-6, PTGS2, and IGF-1, were found in all three clusters, while MMP-9 was found in Go: 0009725 and Go: 0006954, and APOE was found in Go: 0010817 and Go: 0006954. In addition to being included in hormone response and regulation as shown in Figure 4B, the five genes contributed to “inflammatory response” in GO enrichment.

Bio-informatics has built a bridge to illustrate the anti-proliferative effects of chelerythrine. In GEO and GO analyses, factors IL-6, PTGS2, MMP-9, IGF-1, and APOE were proposed. IL-6 and PTGS2 are well-known factors mediating inflammation, while MMP-9 is helpful with the metastasis of gastric cancer (Mroczko et al., 2009). Moreover, activating IGF-1 might suppress inflammation through the PI3K/AKT pathway (Lin et al., 2020a). Despite the lack of evidence for the influence of APOE on inflammation, four of the five genes are supported by evidence from published literature. Interestingly, the four genes consequently activate the classic pathways in gastric cancer, PI3K/AKT and MAPK (Liu et al., 2014; Lin et al., 2020a; Ma et al., 2022). These results seem to point to a correlation between hormone signaling pathways and inflammation in gastric cancer cells.

### 3.6 Chelerythrine mediated hormone signaling pathways

Network pharmacology is ideally suitable to efficiently discover valuable signaling pathways in order to tentatively shed light on the mechanism of chelerythrine for anti-gastric cancer effects. After searching “chelerythrine” in the “TCMSP” database, potential targets were obtained. The term “gastric cancer” was used to obtain potential disease genes from “UniProt.” The six common genes between “gastric cancer” and “chelerythrine” were found to be PTGS2, nuclear receptor coactivator 2 (NCOA2), prostaglandin G/H synthase 1 (PTGS1), potassium voltage-gated channel subfamily H member 2 (KCNH2), retinoic acid receptor RXR-alpha (RXRA), and the mRNA of PKA catalytic subunit c-alpha (PRKACA). The correlations of these genes were predicted using STRING (Figure 4E). Pathways possibly mediated by chelerythrine are shown in Figure 4F. Three hormone-related pathways, thyroid hormone (TH) signaling pathway, estrogen signaling pathway, and oxytocin signaling pathway, were observed and were underlined for their anti-proliferative effects on gastric cancer cells. Thus, we might hypothesize that the inflammatory inhibitor, chelerythrine, might exhibit anti-proliferative effects on gastric cancer cells by mediating hormone-related pathways.

### 3.7 Reviewing cross-regulations of dual COX-2/5-LOX inhibitors on estrogen, thyroid, and oxytocin pathways tentatively

**FIGURE 5:**
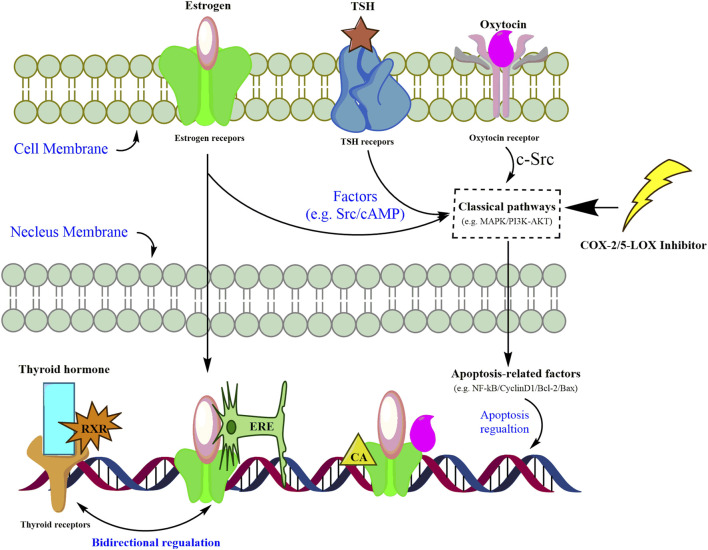
Assumed pathways of estrogen signaling, thyroid signaling, and oxytocin signaling mediated by inflammatory inhibitors in carcinoma cells.

Enrichment of both GO and KEGG indicated that hormone signaling pathways could be mediated by inflammation in gastric cancer cells. However, cross-networks between these pathways and anti-inflammatory candidates are not attractive enough to be sufficiently stressed. A review may help with generally establishing the interplay between these three hormone-related signaling pathways and inflammatory inhibitors. Through reviewing the published literature, we have tentatively presented the complex mediations of inflammatory inhibitors and hormone-related pathways as follows (Figure 5). Hormones, including thyroid, estrogen, and oxytocin, trigger targets in intervening in the dysfunction of nucleus genomic transcription, membrane-initiated signaling, and ligand-independent signaling in carcinoma cells (Zane et al., 2014). Mitogen-activated protein kinase (MAPK) and phosphoinositide 3-kinase (PI3K) are the most reported pathways participating in the proliferation, differentiation, and apoptosis of gastric cancer cells (Zane et al., 2014; Saif and Jiang, 2016; Devost et al., 2022). Once combined with corresponding receptors on cell membranes, these hormones could activate the signaling cascade via mediating MAPK and PI3K pathways, which would further trigger the apoptosis factors and gene transcription in the nucleus (Derwahl and Nicula, 2014; Zane et al., 2014; Saif and Jiang, 2016; Liu et al., 2020). It is also reported that inhibitors of COX-2 or 5-LOX might contribute to the suppression of gastric cancer cells by silencing or calling back MAPK and PI3K/AKT (Kim et al., 2009; Cheng et al., 2014; Liu et al., 2014; Lin et al., 2021). For nucleus gene transcription, the estrogen–receptor complex combines with an estrogen-responsive element (ERE) to locate and promote target genes’ transcription (Zane et al., 2014). Co-activators (CAs), SRC-1 and CBP, are recruited along with the estrogen–receptor complex to enhance the transcription of oxytocin genes (Acevedo-Rodriguez et al., 2015). Due to the lack of thyroid receptors in the carcinoma cell membrane, the thyroid hormone combines with thyroid receptors and RXR to activate the promotor in thyroid receptor element (TRE) sequences in the nucleus (Zane et al., 2014). Given the similarity between the ERE and TRE in nucleotide sequences, the bidirectional regulation of thyroid and estrogen transcription is also listed (Zane et al., 2014). The network shown in Figure 5 roughly summarizes the cross-regulation between these hormone signaling pathways and inflammatory inhibitors. Regardless of the bidirectional regulations between thyroid and estrogen transcription, the mediation of hormones up- and downstream of these three pathways already made the regulation complex and hard to illustrate clearly in a study (Zane et al., 2014; Saif and Jiang, 2016). Supervision of hormone targets in pathways, including the estrogen, thyroid, and oxytocin pathways, might simplify the investigation of mechanisms and contribute to a better understanding of the effects attributed to chelerythrine adoption.

### 3.8 Chelerythrine called back the dysfunctions in the thyroid signaling pathway

In the thyroid signaling pathway, the thyroid-stimulating hormone (TSH) combines with the TSHR to promote the secretion of TH (Gauthier et al., 2020). The TSHR is overexpressed in breast, ovarian, liver, and lung cancer cells (Gauthier et al., 2020). The TH participates in multiple human physiology regulations, such as growth and metabolism, via targeting the thyroid hormone receptors (TRs) (Gauthier et al., 2020). TRs, including TRα1, TRα2, TRα3, TRβ1, and TRβ2, could activate a signaling cascade by associating nuclear receptors, such as RXR (Brown et al., 2013). Upregulated TRα might lead to less distant metastasis and incidence by eliminating risk factors in gastric cancer (Brown et al., 2013). TRα3 is even considered a tumor suppressor and suggested as the target to develop specific agonists (Buzzard et al., 2000). Meanwhile, TRβ1 is downregulated in hepatoma and breast cancer cells and is also considered a suppressor in tumor metastasis based on *in vitro* and *in vivo* experiments (Lin et al., 2020b). Moreover, TRs could form a heterodimer with RXR as TRs/RXR, which further activates the downstream apoptotic gene transcription (Yamazaki et al., 2007; Liu et al., 2019). Thus, typical but important genes and proteins, TSHR, TRα3, TRβ1, and RXR, are selected, the transcription and expression of which are detected to explore the anti-gastric cancer mechanism of chelerythrine.

**FIGURE 6:**
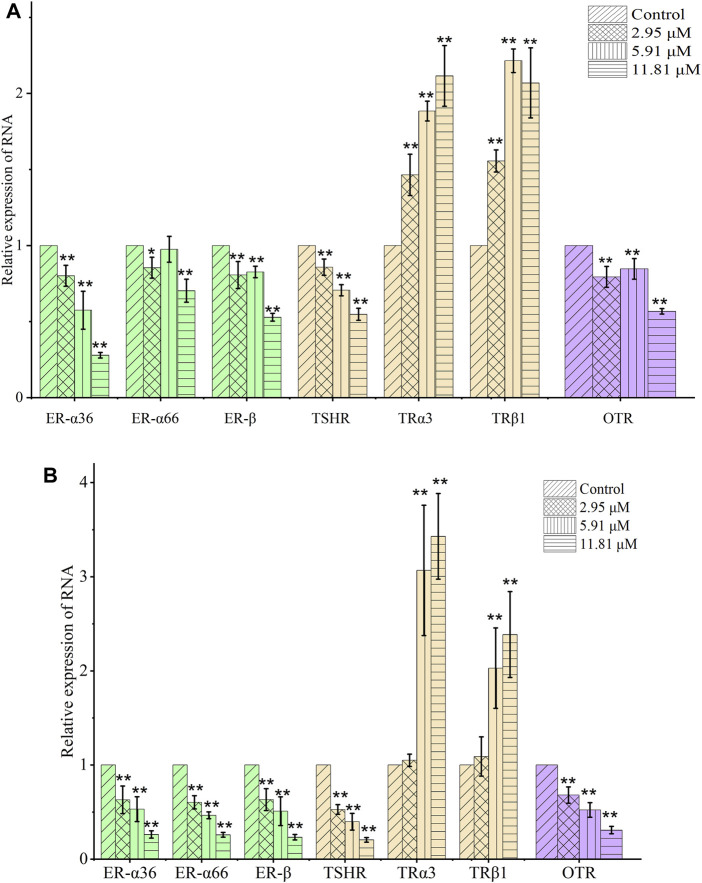
Chelerythrine regulated the expression of mRNA in AGS **(A)** and SGC-7901 **(B)** cells. After treatment with chelerythrine at 0, 2.95, 5.91, and 11.81 μΜ, cells were used in real-time PCR assay. Transcription of genes, including ER-α36, ER-α66, and ER-β, in estrogen pathways (green); TSHR, TRα3, and TRβ1 in thyroid pathways (brown); and OTR in oxytocin pathways (purple) was detected. Data on mRNA are presented as means ± SD. Significance was statistically analyzed by one-way ANOVA. *p < 0.05 and **p < 0.01 vs. control.

**FIGURE 7:**
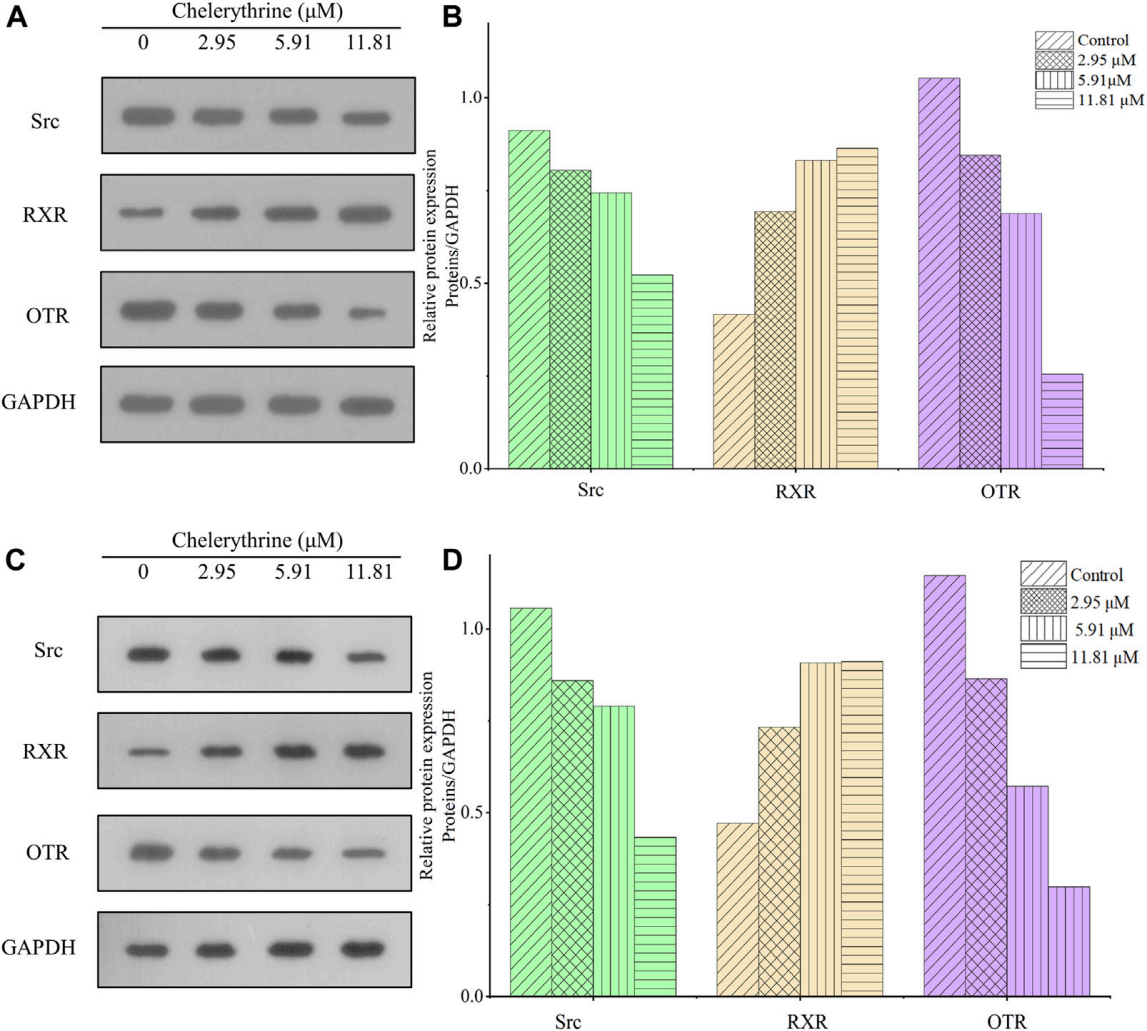
Chelerythrine regulated expression in Western blot assay. **(A,C)** Images of Src (green), RXR (brown), ORT (purple), and GAPDH for AGS and SGC-7901 cells. **(B,D)** Relative expression of RXR, ORT, and Src compared with GAPDH for AGS and SGC-7901 cells. Gray values of GAPDH were used to normalize data and calculate the relative expression of proteins.

For cells being treated with chelerythrine at concentrations of 0, 2.95, 5.91, and 11.81 μM, the transcription and expression of the key targets are shown in Figure 6 and Figure 7. In the thyroid signaling pathway, transcription of the TSHR was significantly reduced (*p* < 0.01) in a dose-dependent manner, while TRα3 and TRβ1 significantly increased at 5.91 and 11.81 μM for AGS and SGC-7901 (*p* < 0.01). RXR was observed to be upregulated. The results, i.e., downregulated TSHR and RXR and upregulated TRα3 and TRβ1, were consistent with enhancing the effects in the thyroid signaling pathway toward anti-gastric cancer cells. As the screened COX-2/5-LOX inhibitor from QAs, chelerythrine might exhibit anti-proliferative potential through the call-back of the thyroid pathway, which was generally attributed to inflammation regulation.

### 3.9 Chelerythrine suppressed ER-α36-Src in estrogen signaling pathway

In the estrogen signaling pathway, estrogen receptors (ERs), including ER-α36, ER-α46, ER-α66, and ER-β, are responsible for the biological process of various cancers (Wang, 2013). Compared with its expression in the adjacent tissue, ER-α36 is detected to be highly expressed and regarded as a promotor of invasion, proliferation, and lymph node metastasis in gastric cancer cells via enhancing the c-Src signaling pathways (Wang et al., 2013). In addition to inhibition of proliferation, overexpressed ER-α66 might accelerate apoptosis and cell cycle arrest of carcinoma cells (Saif and Jiang, 2016). Contrarily, positive expression of ER-α66 at the mRNA level indicates poorer overall survival (Saif and Jiang, 2016). Regarding ER-β, it is deduced as a protector against invasiveness by competing with the transcription of ER-α in gastric cancer cells (Saif and Jiang, 2016). Thus, the detection of mainly genes and important proteins, ER-α36, ER-α66, ER-β, and Src, may help in understanding the apoptotic promotion of chelerythrine in gastric cancer cells.

As shown in Figure 6 and Figure 7, different from the significant inhibition in a dose-dependent way for ER-α36 (*p* < 0.01), chelerythrine showed its increasing and then decreasing inhibition for ER-α66 and ER-β for AGS cells. For SGC-7901, chelerythrine reduced the transcription of ER-α36, ER-α66, and ER-β (*p* < 0.01) dose-dependently. Expression of Src, as shown in Figure 7, was detected to decrease dose-dependently. Overall, the transcription and expression of ER-α36, ER-α66, ER-β, and Src were downregulated dose-dependently when AGS and SGC-7901 cells were treated with chelerythrine. Taking the dysfunction of these genes and targets in gastric cancer cells into consideration, the downregulation of ER-α36-Src was useful for anti-AGS cells, while the protective benefits from ER-α66 and ER-β were weakened. The converse efficacies of the regulations made it difficult to point out the inhibitory or protective effects on AGS cells in the estrogen signaling pathway. As reported in the literature, dysfunction in modulating estrogen receptors might result in higher MMP-2 activity in promoting invasiveness (Rubio et al., 2018). However, the anti-invasion of chelerythrine was observed to be significant in our previous study (Tian et al., 2022). The anti-proliferative and anti-invasive effects of ER-α36 were not competitively reversed by ER-α66 and ER-β. Given the above evidence of chelerythrine, the ERα:ERβ ratio should be further investigated to better illustrate the cumulative and anti-invasive effects on carcinoma cells. Based on the limited clinicopathological and prognostic significance conditions due to the extremely low level of ER-β (Saif and Jiang, 2016), we believe that ER-α36-Src was more effective than ER-α66 and ER-β in gastric cancer cells (Wang et al., 2013). Thus, ER-α36-Src in the estrogen signaling pathway was worthy to be proposed and verified for further anti-gastric cancer study.

### 3.10 Chelerythrine downregulated OTR in the oxytocin signaling pathway

As a G-coupled transmembrane receptor, OTR (only one isoform) at the mRNA and protein levels is extensively reported in neoplastic and non-plastic tissues (Cassoni et al., 2004). Oxytocin could bind to the OTR to downregulate NF-kB, PI3K, cyclin D1, and Bcl-2, which might result in the death of malignant breast cancer cells via inhibiting the effects of ER-α (Khori et al., 2018). For gastric cancer, inhibiting bromodomain-containing protein 9 (BRD9) is attributed to protein mediation in the oxytocin signaling pathway, including calcium voltage-gated channel auxiliary subunit alpha 2 delta 4 (CACNA2D4), calmodulin-like 6 (6CALML6), guanine nucleotide-binding protein (G protein), alpha activating activity polypeptide O (GNAO1), and potassium inwardly-rectifying channel subfamily J, member 5 (KCNJ5) (Wang et al., 2020). Due to the upregulated KCNJ5 and downregulated CACNA2D4, CALML6, and GNAO1, the expression of BRD9 might be suppressed, which might benefit the secretion of oxytocin and further result in the apoptosis of gastric cancer cells (Wang et al., 2020). The OTR is also mentioned as a worthy target for breast cancer (Liu et al., 2020). Finally, the transcription and expression of the OTR in gastric cancer cells might provide evidence to understand the anti-proliferative effects of chelerythrine. At last, the OTR shown in Figure 6 was downregulated significantly (*p* < 0.01), the expression of which was also decreased, as shown in Figure 7.

The unexpectedly reduced regulation of the OTR might result in reduced generation and secretion of oxytocin, which leads to negative, even opposite, effects on anti-gastric cancer cells. Chelerythrine could hardly contribute to suppressing AGS and SGC-7901 via this pathway. The expression of the OTR was not only regulated by factors in the oxytocin pathway but also by factors in the estrogen signaling pathway, e.g., ER-α66 and ER-β. Although the effects on ER-α36 were disclosed for anti-gastric cancer cells, how oxytocin secretion was stimulated via the estrogen signaling pathways is still worthy of further study to illustrate the comprehensive anti-proliferative effects of chelerythrine.

## 4 Conclusion

In this study, COX-2 and 5-LOX enzymes were selected as targets and subjected to ultrafiltration to efficiently screen dual inhibitors. As a result, chelerythrine was proposed as the most prominent bio-active alkaloid among QAs in consideration of enrichment calculation, molecular docking, and anti-proliferative analysis. Molecular docking also suggested that both keeping skeleton atoms in a plane and adding more polar moieties were needed to enhance the anti-proliferative efficacy of chelerythrine for further molecular structure modification. To explore the potential mechanism, bio-informatics and the following review were inadvertently applied, which provided us an opportunity to draw hypothetical cross-interactions and expose the crucial role of hormone-related pathways mediated by inflammatory inhibitors. Further results verified that calling back ER-α36-Src, TSHR, TRα3, TRβ1, and RXR provided an insight into the estrogen pathway and thyroid pathway, which might contribute to the possible inhibition from chelerythrine treatment. Contrarily, regulations of ER-α66, ER-β, and OTR showed conflicting roles in promoting the proliferation of gastric cancer cells owing to the estrogen pathway and oxytocin pathway. Overall, we could tentatively propose that the inflammatory inhibitor (chelerythrine) might mediate anti-proliferative effects on gastric cancer cells through comprehensive cross-regulation of thyroid, estrogen, and oxytocin pathways. Yet further efforts may be helpful in providing direct evidence to understand the hormonal regulatory pathways mediated by inflammatory inhibitors.

## Data Availability

The datasets presented in this study can be found in online repositories. The names of the repository/repositories and accession number(s) can be found in the article/Supplementary Material.

## Abbreviations

COX-2, cyclooxygenase 2; 5-LOX, 5-lipoxygenase; QAs, quaternary alkaloids; GEO, Gene Expression Omnibus; GO, Gene Ontology; KEGG, Kyoto Encyclopedia of Genes and Genomes; TSHR, thyroid-stimulating hormone receptor; TRα3, thyroid Hormone α3; TRβ1, thyroid hormone receptor β1; ER-α36, estrogen receptor α36; ER-α66, estrogen receptor α66; ER-β, estrogen receptor β; OTR, oxytocin receptor; RXR, retinoid X receptor; EFs, enrichment factors; DEGs, differently expressed genes.
